# Self-reported sexually transmitted infections and related symptoms among women in Ghana

**DOI:** 10.1371/journal.pone.0348770

**Published:** 2026-06-24

**Authors:** Florence Gyembuzie Wongnaah, Khadijat Adeleye, Augustus Osborne, Richard Gyan Aboagye, Bright Opoku Ahinkorah

**Affiliations:** 1 Abuakwa North Municipal Health Directorate, Ghana Health Service, Kukurantumi, Eastern Region, Ghana; 2 Elaine Marieb College of Nursing, University of Massachusetts, Amherst, Massachusetts, United States of America; 3 Institute for Development, Western Area, Freetown, Sierra Leone; 4 Department of Family and Community Health, Fred N. Binka School of Public Health, University of Health and Allied Sciences, Hohoe, Ghana; 5 REMS Consultancy Services Limited, Takoradi, Sekondi-Takoradi, Ghana; 6 College of Health, Adelaide University, Adelaide, South Australia, Australia; Menzies School of Health Research: Charles Darwin University, AUSTRALIA

## Abstract

**Introduction:**

Sexually transmitted infections (STIs) remain a major public health issue worldwide, particularly in low- and middle-income countries. Among women, STIs are associated with an elevated risk of HIV infection, infertility, and pelvic inflammatory diseases. This study examined the prevalence of self-reported STIs and related symptoms and their associated factors among women in Ghana.

**Methods:**

Data from the 2022 Ghana Demographic and Health Survey was used for the study. A weighted sample of 12,997 women of reproductive age (15–49 years) was included in the analysis. Four outcome variables were analysed: (1) any STIs, (2) genital discharge, (3) genital sores or ulcers, and (4) a composite measure of any STIs or genital discharge or genital sores/ulcers (STIs and related symptoms). Multivariable binary logistic regression was used to identify the factors associated with self-reported STIs and related symptoms, with results presented as adjusted odds ratios (aORs) and 95% confidence intervals (CIs). The analysis accounted for sampling weights, clustering, and stratification. All analyses were performed in Stata version 17.0.

**Results:**

The prevalence of self-reported any STIs, genital sores or ulcers, and genital discharge among women in Ghana was 6.4% (95% CI: 5.8–7.1), 9.1% (95% CI: 8.3–10.0), and 21.9% (95% CI: 20.6–23.4), respectively. An estimated 26.7% (95% CI: 25.2–28.2) of women reported STIs and related symptoms. Women aged 40–44 (aOR = 0.70, 95% CI: 0.52–0.93) and 45–49 (aOR = 0.55, 95% CI: 0.40–0.76) had lower odds of self-reporting STIs and related symptoms than those aged 15–19. Women who first had sex at age 17 or older (aOR = 0.81, 95% CI: 0.72–0.91) also had lower odds of self-reporting STIs and related symptoms than those who first had sex at age 16 or younger. Additionally, women with one (aOR = 0.73, 95% CI: 0.61–0.87), two (aOR = 0.68, 95% CI: 0.55–0.85), three (aOR = 0.71, 95% CI: 0.56–0.90), and four or more children (aOR = 0.61, 95% CI: 0.48–0.77) had lower odds of self-reporting STIs and related symptoms compared with those without children. Women who were cohabiting (aOR = 0.81, 95% CI: 0.67–0.98) and those previously married (aOR = 0.78, 95% CI: 0.62–0.98) had lower odds of self-reporting STIs and related symptoms than those who had never been in a union. Conversely, women who engaged in multiple sexual partnerships (aOR = 1.95, 95% CI: 1.48–2.58) and those who visited the health facility in the past 12 months (aOR = 1.21, 95% CI: 1.08–1.35) had higher odds of self-reporting STIs and related symptoms compared with those without multiple sexual partnerships and those who did not visit the health facility in the past year, respectively. Compared with women from the Western region, those from Ahafo (aOR = 1.62, 95% CI: 1.28–2.05) and Northern (aOR = 1.82, 95% CI: 1.45–2.28) regions had higher odds of self-reporting STIs and related symptoms.

**Conclusion:**

Self-reported STIs and related symptoms are prevalent among women of reproductive age in Ghana, with variations across the regions. Factors associated with self-reported STIs and related symptoms were women’s age, parity, age at first sex, marital status, number of sexual partners, and health facility visits. Targeted sexual health education focusing on high-risk sexual behaviours, as well as region-specific interventions, are needed to help prevent the incidence of STIs and related symptoms.

## Introduction

Sexually transmitted infections (STIs) remain a major public health concern, transmitted primarily through sexual contact, including vaginal, anal, or oral sex [1]. The World Health Organization (WHO) indicated that more than 1 million new STIs are acquired each day [2]. In 2020 alone, an estimated 374 million new infections were reported among individuals aged 15–49, including 129 million cases of chlamydia, 82 million of gonorrhoea, 7.1 million of syphilis, and 156 million of trichomoniasis [2], resulting in 2.5 million deaths annually from HIV, viral hepatitis, and STIs [3]. STIs impose a disproportionate burden on low- and middle-income countries, with sub-Saharan Africa among the most affected regions [4].

Women, in particular, face unique burden related to STIs due to a combination of anatomical, socio-cultural, and economic factors. STIs can have severe consequences on female reproductive health, leading to infertility, increased vulnerability to HIV, and adverse pregnancy outcomes [2,5]. Many STIs are asymptomatic, which complicates early detection and timely treatment [2]. When STIs are left untreated, they can cause permanent infertility, increase the risk of cancers, and facilitate HIV transmission [5]. It also imposes significant health and economic burdens globally [6]. Surveillance of STIs is therefore crucial for understanding their incidence, distribution, and associated risk factors within communities; however, traditional surveillance methods, which mainly rely on clinical diagnoses, have notable limitations, including underreporting, stigma, and limited access to health care [7]. These factors make it difficult to accurately assess the true burden and determinants of STI transmission.

Several studies have examined the prevalence and determinants of self-reported STIs among women. For example, the prevalence of self-reported STIs among women of reproductive age in Ethiopia was 3.0%, with significant associations with age, marital status, education, history of pregnancy termination, and risky sexual behaviours [8]. In Mali, a higher prevalence of 14.1% was observed among adolescent girls and young women, linked to socioeconomic status, age, and education [9]. Also, Stewart et al. [10] emphasised that sub-Saharan Africa bears the highest burden of STIs [10], which could be attributed to several factors, including late detection, limited screening services, and the associated costs.

In Ghana, a trend analysis study conducted in the Ho Municipality showed that HIV and syphilis infections were more prevalent in the asymptomatic population than in pregnant women [11]. Although data from the Ghana Demographic and Health Survey (GDHS) and related studies report relatively low STIs prevalence among young women, ranging from 0.3% to about 7% [12,13], the regional burden remains substantial. A 2020 systematic review estimated chlamydia prevalence among females aged 15–49 in sub-Saharan Africa at 6.6% [14]. However, studies on STIs in Ghana have largely focused on specific populations or contextual factors, such as biological prevalence in selected settings and transmission dynamics [15,16], with some focusing exclusively on men [17], limiting the applicability of the findings to women of reproductive age. Despite this growing body of evidence, there remains limited recent nationally representative evidence using the 2022 GDHS to assess self-reported any STIs and related symptoms among women in Ghana, particularly with respect to regional and behavioural correlates. This gap limits a comprehensive understanding of the current burden and determinants of STIs among women of reproductive age in Ghana.

This study therefore seeks to address these knowledge gaps by examining the prevalence and correlates of self-reported STIs and related symptoms among women of reproductive age in Ghana. Specifically, it aims to estimate the prevalence of self-reported STIs and related symptoms, as well as to identify associated socio-demographic and behavioural factors. The findings will inform the development of context-specific, evidence-based sexual health interventions in Ghana and similar settings.

## Methods

### Data source

Our study utilised data from the 2022 GDHS. We extracted the data from the women’s recode file for analysis. The GDHS is part of the global DHS programme that collects demographic and health data on men, women, and children worldwide [18,19]. Since the inception of the DHS, over 90 low- and middle-income countries (LMICs) have participated in more than 350 surveys [18]. The GDHS employed a cross-sectional design [19]. Respondents were sampled using a multistage sampling design. Structured questionnaires were administered by trained data collectors to gather data from respondents. The detailed methodology used in the 2022 GDHS, including design, sampling, and data collection techniques, is available in the final report compiled by the Ghana Statistical Service (GSS) and ICF International [19], as well as in other standard DHS reports [18,20]. The 2022 GDHS interviewed 15,014 women aged 15–49 years. After excluding observations with missing data on variables included in the analysis, the final analytic sample was 12,997 women. This represented approximately 86.6% of eligible women.

### Variables

Four outcome variables were considered in this study. The variables consisted of the presence of (i) genital discharge, (ii) genital sore/ulcer, (iii) any STIs (self-reported STIs not restricted to specific symptoms such as abnormal genital discharge or genital sores/ulcers), and (iv) any STIs, genital discharge or genital sore or ulcer (STIs and related symptoms). In the DHS questionnaire, the women were asked the questions, “During the last 12 months, have you had a disease which you got through sexual contact?”, “During the last 12 months, have you had a bad-smelling abnormal genital discharge?”, and “During the last 12 months, have you had a genital sore or ulcer?” [18,19]. The response options for each question were yes, no, and don’t know. We recoded the yes option as “1=yes”. Women who responded “no” and “don’t know” were merged to form the “no” category for each variable and were coded as “0=no”. A composite outcome was created to capture women experiencing any of the STIs or related symptoms, improving sensitivity in identifying potential infections in settings where laboratory confirmation is unavailable.

Sixteen exposure variables were included in the study. The variables were selected based on their availability in the GDHS and their association with STIs and related symptoms in the literature [9,21–23]. The variables were age of the women, level of education, marital status, current working status, parity (number of children), exposure to mass media, religion, health insurance coverage, visit to health facility in the last 12 months, ever tested for HIV, age at first sexual intercourse, multiple sexual partnerships, condom use during sex with recent partner, wealth index, place of residence, and region. Table 1 contains the detailed categories of each variable included in the study.

**Table 1:** Background characteristics of the women.

Variables	Weighted sample	Weighted percentage (%)
**Women’s age (years)**		
15-19	1,057	8.1
20-24	2,339	18.0
25-29	2,272	17.5
30-34	2,246	17.3
35-39	2,073	15.9
40-44	1,688	13.0
45-49	1,321	10.2
**Level of education**		
No education	2,383	18.3
Primary	1,853	14.3
Secondary	7,413	57.0
Higher	1,348	10.4
**Marital status**		
Never in union	3,163	24.3
Married	6,061	46.6
Cohabiting	2,217	17.1
Previously married	1,556	12.0
**Religion**		
Christianity	9,988	76.8
Islam	2,469	19.0
African Traditional religion	258	2.0
No religion or other	282	2.2
**Current working status**		
Not working	2,489	19.2
Working	10,508	80.8
**Number of children**		
None	2,745	21.1
One	2,443	18.8
Two	2,023	15.6
Three	1,780	13.7
Four or more	4,006	30.8
**Covered by health insurance**		
No	1,215	9.3
Yes	11,782	90.7
**Visited health facility in last 12 months**		
No	5,759	44.3
Yes	7,238	55.7
**Exposed to mass media**		
No	1493	11.5
Yes	11504	88.5
**Ever tested for HIV**		
No	4,452	34.3
Yes	8,545	65.7
**Condom use during last sex with most recent partner**		
No	12,514	96.3
Yes	483	3.7
**Multiple sexual partnerships**		
No	12,668	97.5
Yes	329	2.5
**Age at first sex**		
16 years and below	5,065	39.0
17 years and above	7,932	61.0
**Wealth index**		
Poorest	2,190	16.9
Poorer	2,383	18.3
Middle	2,730	21.0
Richer	2,929	22.5
Richest	2,765	21.3
**Place of residence**		
Urban	7,267	55.9
Rural	5,730	44.1
**Region**		
Western	826	6.4
Central	1,458	11.2
Greater Accra	1,943	14.9
Volta	597	4.6
Eastern	1053	8.1
Ashanti	2564	19.7
Western North	371	2.8
Ahafo	280	2.2
Bono	484	3.7
Bono East	607	4.7
Oti	360	2.8
Northern	1027	7.9
Savannah	281	2.2
North East	260	2.0
Upper East	549	4.2
Upper West	335	2.6

### Statistical analyses

We used Stata version 17.0 (StataCorp, College Station, TX, USA) to perform all the statistical analyses. The analyses were carried out in four stages. In the first stage, we cleaned the data by dropping all missing observations, recoding variables to form meaningful categories, and weighting the data per DHS guidelines [18]. The weighting accounted for the complex DHS survey design, including sampling weights, clustering, and stratification. The second stage involved presenting the background characteristics of the respondents using frequencies and percentages. We also estimated the prevalence of STIs and related symptoms as percentages, with corresponding confidence intervals (CIs). In the third stage, prior to model estimation, multicollinearity among the explanatory variables was assessed using the variance inflation factor, and no evidence of collinearity was found. We performed a bivariate analysis to examine the distribution of STIs and related symptoms across respondents’ background characteristics. Exposure variables with a p-value <0.05 in the bivariate analysis were included in multivariable logistic regression models. In the final stage, we performed a survey-weighted multivariable binary logistic regression to examine the factors associated with STIs and related symptoms. Separate models were fitted for each outcome variable (i.e., any STIs, genital discharge, genital sores/ulcers, and any STIs and related symptoms) to ensure comparability across models and to control for potential confounding. The results were presented using adjusted odds ratios (aORs) with their respective 95% CIs. Statistical significance was set at *p* < 0.05.

### Ethical consideration

This study did not require additional ethical approval because we used publicly available, anonymised secondary data from the DHS Program. We obtained permission from the Monitoring and Evaluation to Assess and Use Results Demographic and Health Surveys (MEA-SURE DHS) Program to access and use the data for our study. Ethical approval was granted by the ICF Institutional Review Board, with reference number 45 CFR 46. To comply with the local ethical standards, ethical approval was also sought from the Ghana Health Service Ethics Review Committee before commencing the survey.

## Results

### Background characteristics of the women

Table 1 presents the background characteristics of women in Ghana. A total of 18.0% of women were aged 20–24. More than half of the women had secondary education (57.0%), and nearly half were married (46.6%). More than three-quarters of women were Christians (76.8%), and the majority were working (80.8%), covered by health insurance (90.7%), and exposed to mass media (88.5%). Over half of the women had visited a health facility in the last 12 months (55.7%), and 65.7% had ever tested for HIV, while 55.9% resided in urban areas. Additionally, 3.7% of women used condoms during sex with their most recent partner, 2.5% had multiple sexual partnerships, 61.0% had their first sexual intercourse at age 17 or older, and 22.5% lived in households in the richest wealth quintile.

### Prevalence of STIs and related symptoms

**Fig 1:**
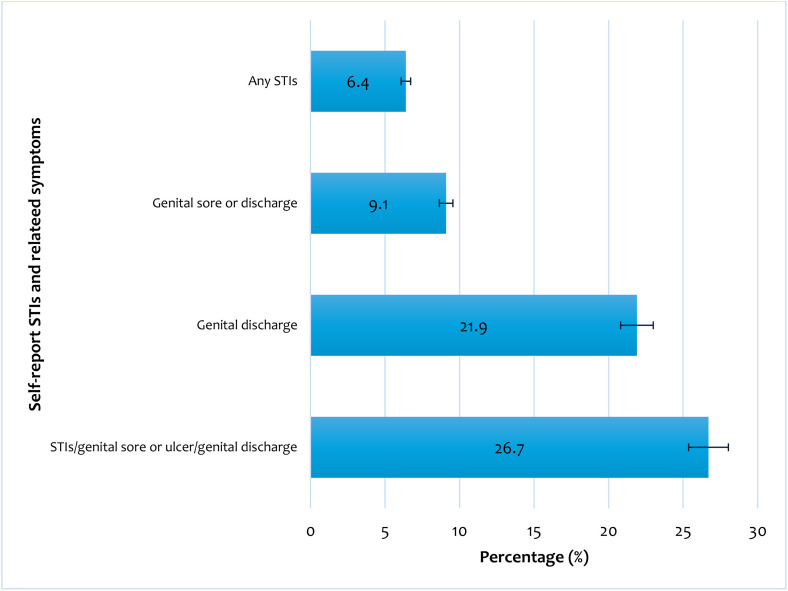
Prevalence of self-reported STIs and related symptoms among women in Ghana.

Fig 1 shows the prevalence of self-reported STIs and related symptoms among women in Ghana. An estimated 26.7% (95% CI: 25.2–28.2) of women reported any STIs and related symptoms. For individual components, the highest prevalence was observed for genital discharge at 21.9% (95% CI: 20.6–23.4), followed by genital sore or ulcer at 9.1% (95% CI: 8.3–10.0). The lowest prevalence was observed for any self-reported STIs at 6.4% (95% CI: 5.8–7.1).

### Bivariate results of self-reported STIs and related symptoms among women in Ghana

Tables 2 and 3 present the bivariate analysis of self-reported STIs, related symptoms, and their composite measure (STIs and related symptoms). Women aged 15–19 reported the highest prevalence of STIs and related symptoms among all age groups (37.6%), followed by women aged 20–24 (33.6%). Women with higher education recorded the highest prevalence of STIs and related symptoms among educational subgroups (27.6%). Women who had never been in a union also had the highest prevalence (35.2%), while women belonging to traditional religions recorded the highest prevalence of STIs and related symptoms (34.7%). Those who were not working reported a slightly higher prevalence (29.8%). Prevalence was also higher among women who used condoms at last sex (33.9%). Additionally, women with no children had the highest prevalence of STIs and related symptoms (36.5%), whereas those who visited a health facility in the last 12 months had the highest prevalence of STIs and related symptoms (27.9%). STIs and related symptoms were more common among women who had never tested for HIV (28.6%). The prevalence of STIs and related symptoms was highest among women with multiple sexual partners (47.1%), while women who had their first sexual contact at age 16 or younger had a prevalence of 28.9%. Among wealth categories, women in the middle quintile had the highest prevalence (28.9%) of STIs and related symptoms. By region, the Northern region recorded the highest prevalence (38.9%), followed by Ahafo (37.8%) and Savannah (30.2%).

**Table 2:** Bivariable analysis of self-reported genital discharge and genital sores/ulcers.

Variables	Genital sores/ ulcers		Genital discharge	
	% [95% CI]	p-value	% [95% CI]	p-value
**Women’s age (years)**		<0.001		<0.001
15-19	12.1 [9.9, 14.8]		31.9 [28.5, 35.6]	
20-24	11.1 [9.4, 13.0]		28.8 [26.1, 31.6]	
25-29	10.4 [8.9, 12.1]		24.3 [21.8, 27.0]	
30-34	8.6 [7.1, 10.4]		20.7 [18.4, 23.2]	
35-39	8.3 [6.9, 10.1]		18.2 [15.8, 21.0]	
40-44	7.3 [5.9, 9.0]		16.5 [14.3, 18.9]	
45-49	5.6 [4.2, 7.3]		12.8 [10.5, 15.5]	
**Level of education**		0.016		0.121
No education	11.3 [9.5, 13.5]		21.2 [18.4, 24.3]	
Primary	8.1 [6.7, 9.7]		19.2 [16.9, 21.8]	
Secondary	9.0 [8.0, 10.1]		22.8 [21.2, 24.4]	
Higher	7.4 [5.5, 9.9]		22.3 [19.2, 25.8]	
**Marital status**		0.054		<0.001
Never in union	10.5 [9.0, 12.2]		30.6 [28.4, 33.0]	
Married	9.0 [8.0, 10.2]		19.8 [18.1, 21.7]	
Cohabiting	9.0 [7.2, 11.1]		19.6 [17.1, 22.3]	
Previously married	7.1 [5.5, 9.0]		15.9 [13.7, 18.4]	
**Religion**		0.004		0.227
Christianity	8.4 [7.5, 9.4]		21.5 [20.1, 23.1]	
Islam	11.4 [9.5, 13.7]		22.1 [19.4, 25.1]	
African Traditional religion	17.7 [9.7, 30.1]		30.1 [17.4, 46.9]	
No religion or other	8.4 [4.4, 15.6]		27.8 [21.8, 34.7]	
**Current working status**		0.001		0.003
Not working	12.1 [10.1, 14.3]		25.2 [22.7, 27.9]	
Working	8.4 [7.6, 9.4]		21.2 [19.7, 22.7]	
**Number of children**		0.002		<0.001
None	11.5 [9.9, 13.2]		31.0 [28.4, 33.7]	
One	8.9 [7.6, 10.5]		23.7 [21.3, 26.3]	
Two	9.1 [7.5, 10.9]		20.1 [17.8, 22.7]	
Three	8.6 [7.0, 10.5]		18.6 [16.2, 21.3]	
Four or more	7.9 [6.8, 9.1]		17.1 [15.3, 19.1]	
**Covered by health insurance**		0.375		0.251
No	8.3 [6.5, 10.5]		23.6 [20.5, 27.0]	
Yes	9.2 [8.4, 10.2]		21.8 [20.4, 23.2]	
**Visited health facility in last 12 months**		0.291		0.117
No	8.7 [7.6, 10.0]		21.0 [19.4, 22.8]	
Yes	9.5 [8.5, 10.6]		22.7 [21.0, 24.4]	
**Exposed to mass media**		0.008		0.333
No	12.1 [9.7, 15.1]		23.6 [20.0, 27.6]	
Yes	8.8 [7.9, 9.7]		21.7 [20.4, 23.2]	
**Ever tested for HIV**		0.002		0.004
No	10.7 [9.3, 12.3]		24.2 [22.0, 26.4]	
Yes	8.3 [7.5, 9.3]		20.8 [19.3, 22.4]	
**Condom use during last sex with most recent partner**		0.121		0.013
No	9.0 [8.2, 10.0]		21.7 [20.3, 23.2]	
Yes	11.7 [8.5, 16.0]		27.6 [22.9, 32.8]	
**Multiple sexual partnerships**		<0.001		<0.001
No	8.7 [7.9, 9.6]		21.5 [20.1, 23.0]	
Yes	25.3 [19.7, 31.9]		37.6 [31.2, 44.5]	
**Age at first sex**		<0.001		<0.001
16 years and below	11.0 [9.7, 12.5]		24.2 [22.3, 26.3]	
17 years and above	7.9 [7.1, 8.8]		20.5 [19.0, 22.0]	
**Wealth index**		0.001		0.675
Poorest	11.3 [9.3, 13.7]		22.7 [19.6, 26.1]	
Poorer	11.3 [9.4, 13.6]		21.9 [19.5, 24.5]	
Middle	8.1 [6.8, 9.7]		20.3 [18.0, 22.8]	
Richer	8.8 [7.3, 10.5]		22.3 [20.0, 24.8]	
Richest	6.9 [5.5, 8.7]		22.7 [19.8, 25.8]	
**Place of residence**		0.111		0.871
Urban	8.5 [7.4, 9.8]		22.1 [20.2, 24.1]	
Rural	9.9 [8.7, 11.3]		21.8 [19.9, 23.9]	
**Region**		<0.001		<0.001
Western	9.4 [6.9, 12.8]		24.7 [20.7, 29.2]	
Central	8.0 [5.6, 11.2]		21.0 [17.4, 25.2]	
Greater Accra	2.3 [1.4, 3.9]		18.5 [14.3, 23.5]	
Volta	6.5 [4.3, 9.7]		17.2 [14.0, 21.0]	
Eastern	9.8 [7.3, 13.0]		26.2 [20.9, 32.3]	
Ashanti	9.6 [7.4, 12.4]		22.4 [19.4, 25.8]	
Western North	7.4 [5.0, 10.8]		23.4 [19.5, 27.8]	
Ahafo	15.3 [10.7,21.3]		32.5 [26.6, 39.0]	
Bono	7.7 [4.7, 12.4]		19.3 [15.5, 23.8]	
Bono East	10.3 [6.9, 15.1]		18.2 [13.1, 24.7]	
Oti	8.5 [5.9, 11.9]		15.7 [12.8, 19.1]	
Northern	23.9 [18.6, 30.1]		32.5 [25.1, 40.8]	
Savannah	15.6 [11.9, 20.1]		25.4 [21.4, 30.0]	
North East	7.2 [4.7, 10.8]		18.4 [14.3, 23.3]	
Upper East	5.5 [3.7, 8.0]		12.4 [8.7, 17.3]	
Upper West	6.4 [4.9, 8.5]		20.8 [17.7, 24.2]	

**Table 3:** Bivariate analysis of self-reported any STIs and STIs and related symptoms.

Variables	Any STIs		STIs and related symptoms	
	% [95% CI]	p-value	% [95% CI]	p-value
**Women’s age (years)**		<0.001		<0.001
15-19	7.1 [5.4, 9.2]		37.6 [34.0, 41.4]	
20-24	8.1 [6.6, 9.8]		33.6 [30.7, 36.5]	
25-29	8.1 [6.8, 9.7]		29.3 [26.7, 32.1]	
30-34	6.7 [5.3, 8.5]		25.2 [22.6, 27.9]	
35-39	5.2 [4.1, 6.7]		23.3 [20.6, 26.2]	
40-44	4.3 [3.2, 5.8]		20.7 [18.3, 23.3]	
45-49	3.8 [2.5, 5.7]		16.8 [14.2, 19.9]	
**Level of education**		0.001		0.148
No education	4.4 [3.7, 5.4]		25.8 [22.8, 29.0]	
Primary	5.3 [4.2, 6.8]		24.0 [21.4, 26.8]	
Secondary	7.2 [6.3, 8.2]		27.5 [25.9, 29.2]	
Higher	6.9 [5.3, 8.9]		27.6 [24.2, 31.3]	
**Marital status**		0.001		<0.001
Never in union	8.3 [7.0, 9.9]		35.2 [32.8, 37.7]	
Married	5.5 [4.8, 6.3]		24.4 [22.5, 26.3]	
Cohabiting	6.8 [5.5, 8.4]		24.8 [22.1, 27.8]	
Previously married	5.3 [3.9, 7.3]		21.2 [18.5, 24.1]	
**Religion**		0.405		0.356
Christianity	6.6 [5.8, 7.4]		26.2 [24.6, 27.8]	
Islam	6.2 [5.1, 7.4]		27.4 [24.5, 30.5]	
African Traditionalist	4.1 [2.2, 7.4]		34.7 [19.7, 53.5]	
No religion or other	4.8 [2.5, 9.3]		31.5 [24.9, 38.9]	
**Current working status**		0.212		0.008
Not working	7.1 [5.9, 8.6]		29.8 [27.1, 32.5]	
Working	6.2 [5.5, 7.0]		26.0 [24.4, 27.6]	
**Number of children**		<0.001		<0.001
None	9.5 [8.0, 11.2]		36.5 [33.9, 39.2]	
One	6.5 [5.3, 7.9]		28.2 [25.7, 30.9]	
Two	6.2 [5.0, 7.7]		25.1 [22.6, 27.8]	
Three	6.5 [5.1, 8.1]		24.2 [21.6, 26.9]	
Four or more	4.3 [3.6, 5.1]		21.0 [19.0, 23.1]	
**Covered by health insurance**		0.366		0.754
No	5.7 [4.3, 7.5]		27.2 [23.8, 30.9]	
Yes	6.5 [5.8, 7.2]		26.6 [25.2, 28.2]	
**Visited health facility in last 12 months**		0.925		0.013
No	6.4 [5.5, 7.5]		25.2 [23.4, 27.1]	
Yes	6.4 [5.6, 7.2]		27.9 [26.1, 29.7]	
**Exposed to mass media**		<0.001		0.673
No	4.1 [3.2, 5.3]		27.5 [23.5, 31.9]	
Yes	6.7 [6.0, 7.4]		26.6 [25.1, 28.1]	
**Ever tested for HIV**		0.778		0.021
No	6.5 [5.5, 7.7]		28.6 [26.3, 31.1]	
Yes	6.3 [5.7, 7.1]		25.7 [24.1, 27.3]	
**Condom use during last sex with most recent partner**		0.007		0.003
No	6.3 [5.6, 6.9]		26.4 [24.9, 27.9]	
Yes	9.9 [7.1, 13.6]		33.9 [28.8, 39.5]	
**Multiple sexual partnerships**		<0.001		<0.001
No	6.1 [5.5, 6.8]		26.2 [24.7, 27.7]	
Yes	16.2 [11.5, 22.2]		47.1 [40.5, 53.9]	
**Age at first sex**		0.442		0.001
16 years and below	6.7 [5.8, 7.7]		28.9 [26.8, 31.1]	
17 years and above	6.2 [5.5, 7.1]		25.3 [23.8, 26.9]	
**Wealth index**		0.022		0.800
Poorest	4.5 [3.7, 5.4]		27.0 [23.6, 30.8]	
Poorer	5.8 [4.6, 7.2]		26.4 [23.9, 29.1]	
Middle	6.7 [5.5, 8.2]		25.3 [22.8, 28.0]	
Richer	7.2 [5.9, 8.9]		27.2 [24.6, 30.0]	
Richest	7.3 [5.9, 9.0]		27.5 [24.5, 30.7]	
**Place of residence**		0.017		0.543
Urban	7.1 [6.1, 8.1]		27.1 [25.1, 29.2]	
Rural	5.5 [4.8, 6.4]		26.2 [24.1, 28.3]	
**Region**		0.137		<0.001
Western	6.4 [4.6, 9.0]		28.1 [24.0, 32.6]	
Central	6.0 [4.3, 8.4]		26.2 [22.2, 30.5]	
Greater Accra	5.8 [4.1, 8.2]		22.3 [17.9, 27.5]	
Volta	4.8 [3.5, 6.7]		21.4 [17.7, 25.6]	
Eastern	5.2 [3.5, 7.5]		29.7 [24.1, 36.0]	
Ashanti	7.6 [5.8, 9.9]		28.0 [24.8, 31.5]	
Western North	7.4 [5.3, 10.2]		27.6 [23.1, 32.5]	
Ahafo	10.0 [7.5, 13.3]		37.8 [32.3, 43.6]	
Bono	7.1 [5.2, 9.6]		23.3 [18.5, 28.9]	
Bono East	5.4 [3.9, 7.5]		23.4 [17.6, 30.4]	
Oti	5.3 [3.9, 7.3]		22.5 [19.4, 25.8]	
Northern	7.3 [5.7, 9.3]		38.9 [31.0, 47.3]	
Savannah	3.7 [2.2, 6.1]		30.2 [25.1, 35.8]	
North East	9.6 [6.9, 13.3]		24.0 [19.7, 28.9]	
Upper East	5.4 [3.6, 8.1]		16.8 [12.2, 22.7]	
Upper West	5.3 [3.3, 8.3]		24.4 [21.0, 28.1]	

### Factors associated with STIs and related symptoms among women in Ghana

#### STIs and related symptoms.

Table 4 presents the factors associated with self-reported STIs and related symptoms. Women aged 40–44 (aOR = 0.70, 95% CI: 0.52–0.93) and 45–49 (aOR = 0.55, 95% CI: 0.40–0.76) had lower odds of self-reporting STIs and related symptoms than those aged 15–19. Women who first had sex at age 17 or older (aOR = 0.81, 95% CI: 0.72–0.91) also had lower odds of self-reported STIs and related symptoms compared with those who first had sex at age 16 or younger. Additionally, women with one (aOR = 0.73, 95% CI: 0.61–0.87), two (aOR = 0.68, 95% CI: 0.55–0.85), three (aOR = 0.71, 95% CI: 0.56–0.90), and four or more children (aOR = 0.61, 95% CI: 0.48–0.77) had lower odds of self-reporting STIs and related symptoms compared with those with no children. Women who were cohabiting (aOR = 0.81, 95% CI: 0.67–0.98) and those previously married (aOR = 0.78, 95% CI: 0.62–0.98) had lower odds of self-reporting STIs and related symptoms than those who had never been in a union. Also, women residing in the Greater Accra (aOR = 0.78, 95% CI: 0.60–0.99), Volta (aOR = 0.72, 95% CI: 0.56–0.94), Bono East (aOR = 0.78, 95% CI: 0.62–0.99), and Upper East (aOR = 0.53, 95% CI: 0.41–0.69) regions had lower odds of self-reporting STIs and related symptoms relative to those who lived in the Western region. Conversely, women engaged in multiple sexual partnerships (aOR = 1.95, 95% CI: 1.48–2.58) and those who visited the health facility in the past 12 months (aOR = 1.21, 95% CI: 1.08–1.35) had higher odds of self-reporting STIs and related symptoms compared with those without multiple sexual partnerships and those who did not visit the health facility in the past 12 months, respectively. Compared with women from the Western region, those from Ahafo (aOR = 1.62, 95% CI: 1.28–2.05) and Northern (aOR = 1.82, 95% CI: 1.45–2.28) regions also had higher odds of self-reporting STIs and related symptoms.

**Table 4:** Factors associated with STIs and related symptoms among women in Ghana.

Variables	Genital sore or ulcer	Genital discharge	Any STIs	Any STIs and related symptoms
	aOR [95% CI]	aOR [95% CI]	aOR [95% CI]	aOR [95% CI]
**Women’s age (years)**				
15-19	1.00	1.00	1.00	1.00
20-24	1.17 [0.88, 1.56]	1.05 [0.85, 1.30]	1.29 [0.90, 1.87]	0.98 [0.79, 1.20]
25-29	1.23 [0.89, 1.71]	0.94 [0.74, 1.18]	1.45 [0.97, 2.17]	0.88 [0.70, 1.10]
30-34	1.07 [0.74, 1.57]	0.82 [0.63, 1.08]	1.32 [0.82, 2.13]	0.78 [0.60, 1.01]
35-39	1.18 [0.79, 1.75]	0.76 [0.57, 1.01]	1.07 [0.66, 1.73]	0.77 [0.59, 1.01]
40-44	1.04 [0.68, 1.59]	0.69^*^ [0.51, 0.95]	0.93 [0.55, 1.56]	0.70^*^ [0.52, 0.93]
45-49	0.77 [0.49, 1.21]	0.52^***^ [0.37, 0.74]	0.82 [0.46, 1.46]	0.55^***^ [0.40, 0.76]
**Religion**				
Christianity	1.00			
Islam	0.88 [0.72, 1.07]			
African Traditional religion	1.07 [0.74, 1.56]			
No religion or other	0.81 [0.45, 1.47]			
**Level of education**				
No education	1.00		1.00	
Primary	0.94 [0.73, 1.21]		0.99 [0.72, 1.38]	
Secondary	1.07 [0.85, 1.35]		1.11 [0.85, 1.46]	
Higher	0.88 [0.59, 1.32]		0.89 [0.59, 1.36]	
**Current working status**				
Not working	1.00	1.00		1.00
Working	0.79^*^ [0.66, 0.94]	1.07 [0.93, 1.22]		1.08 [0.95, 1.24]
**Number of children**				
None	1.00	1.00	1.00	1.00
One	0.76^*^ [0.59, 0.99]	0.79^*^ [0.65, 0.95]	0.66^**^ [0.49, 0.90]	0.73^***^ [0.61, 0.87]
Two	0.79 [0.59, 1.06]	0.72^**^ [0.57, 0.90]	0.64^*^ [0.45, 0.93]	0.68^***^ [0.55, 0.85]
Three	0.81 [0.58, 1.13]	0.71^**^ [0.56, 0.91]	0.74 [0.50, 1.09]	0.71^**^ [0.56, 0.90]
Four or more	0.68^*^ [0.48, 0.96]	0.67^**^ [0.53, 0.86]	0.57^**^ [0.38, 0.86]	0.61^***^ [0.48, 0.77]
**Exposed to mass media**				
No	1.00		1.00	
Yes	1.02 [0.82, 1.25]		1.24 [0.92, 1.67]	
**Ever tested for HIV**				
No	1.00	1.00		1.00
Yes	1.05 [0.87, 1.26]	1.08 [0.95, 1.23]		1.09 [0.97, 1.24]
**Multiple sexual partnerships**				
No	1.00	1.00	1.00	1.00
Yes	3.06^***^ [2.18, 4.29]	1.65^***^ [1.24, 2.19]	2.42^***^ [1.64, 3.58]	1.95^***^ [1.48, 2.58]
**Age at first sex**				
16 years and below	1.00	1.00		1.00
17 years and above	0.72^***^ [0.61, 0.85]	0.79^***^ [0.70, 0.89]		0.81^***^ [0.72, 0.91]
**Wealth index**				
Poorest	1.00		1.00	
Poorer	1.16 [0.94, 1.43]		1.18 [0.89, 1.57]	
Middle	0.86 [0.67, 1.11]		1.23 [0.89, 1.69]	
Richer	1.08 [0.83, 1.41]		1.27 [0.89, 1.82]	
Richest	1.01 [0.73, 1.41]		1.29 [0.87, 1.92]	
**Region**				
Western	1.00	1.00		1.00
Central	0.80 [0.55, 1.16]	0.81 [0.63, 1.05]		0.90 [0.71, 1.14]
Greater Accra	0.24^***^ [0.14, 0.43]	0.73^*^ [0.56, 0.95]		0.78^*^ [0.60, 0.99]
Volta	0.70 [0.46, 1.06]	0.67^**^ [0.51, 0.88]		0.72^*^ [0.56, 0.94]
Eastern	1.14 [0.78, 1.65]	1.17 [0.91, 1.50]		1.16 [0.92, 1.48]
Ashanti	1.11 [0.78, 1.58]	0.92 [0.73, 1.17]		1.04 [0.83, 1.31]
Western North	0.80 [0.53, 1.22]	0.95 [0.74, 1.23]		0.99 [0.77, 1.27]
Ahafo	1.74^**^ [1.22, 2.49]	1.53^***^ [1.20, 1.95]		1.62^***^ [1.28, 2.05]
Bono	0.81 [0.55, 1.21]	0.73^*^ [0.56, 0.95]		0.78 [0.61, 1.00]
Bono East	1.15 [0.80, 1.64]	0.67^**^ [0.52, 0.87]		0.78^*^ [0.62, 0.99]
Oti	0.98 [0.67, 1.43]	0.59^***^ [0.45, 0.78]		0.80 [0.62, 1.02]
Northern	3.57^***^ [2.51, 5.08]	1.59^***^ [1.26, 2.01]		1.82^***^ [1.45, 2.28]
Savannah	2.10^***^ [1.43, 3.09]	1.12 [0.86, 1.45]		1.22 [0.95, 1.57]
North East	0.88 [0.58, 1.35]	0.74^*^ [0.56, 0.97]		0.89 [0.69, 1.14]
Upper East	0.61^*^ [0.40, 0.92]	0.44^***^ [0.33, 0.58]		0.53^***^ [0.41, 0.69]
Upper West	0.76 [0.49, 1.16]	0.86 [0.66, 1.13]		0.90 [0.70, 1.17]
**Marital status**				
Never in union		1.00	1.00	1.00
Married		0.84 [0.70, 1.02]	1.08 [0.80, 1.48]	0.88 [0.73, 1.05]
Cohabiting		0.72^**^ [0.59, 0.89]	1.13 [0.81, 1.57]	0.81^*^ [0.67, 0.98]
Previously married		0.68^**^ [0.53, 0.86]	0.99 [0.65, 1.52]	0.78^*^ [0.62, 0.98]
**Condom use during last sex with most recent partner**				
No		1.00	1.00	1.00
Yes		1.00 [0.77, 1.29]	1.21 [0.83, 1.78]	1.05 [0.82, 1.35]
**Place of residence**				
Urban			1.00	
Rural			0.91 [0.72, 1.15]	
**Visited health facility in last 12 months**				
No				1.00
Yes				1.21^***^ [1.08, 1.35]
***N***	**12,997**	**12,997**	**12,997**	**12,997**
**Pseudo *R*** ^**2**^	**0.072**	**0.039**	**0.023**	**0.037**

**Note**: Blank cells indicate variables not included in the final multivariable model for that outcome. “Any STIs and related symptoms” refers to a composite outcome comprising any STI, genital discharge, or genital sores/ulcers.

#### Any STIs.

Women who had one (aOR = 0.66, 95% CI: 0.49–0.90), two (aOR = 0.64, 95% CI: 0.45–0.93), and four or more children (aOR = 0.57, 95% CI: 0.38–0.86) had lower odds of self-reporting any STIs compared with those with no children. However, women who engaged in multiple sexual partnerships (aOR = 2.42, 95% CI: 1.64–3.58) had higher odds of self-reporting any STIs compared with those without multiple sexual partnerships (Table 4).

#### Genital discharge.

From Table 4, women aged 40–44 (aOR = 0.69, 95% CI: 0.51–0.95) and 45–49 (aOR = 0.52, 95% CI: 0.37–0.74) had lower odds of self-reporting genital discharge compared with those aged 15–19. Women who first had sex at age 17 or older (aOR = 0.79, 95% CI: 0.70–0.89) had lower odds of self-reporting genital discharge than those who first had sex at age 16 or younger. Also, women who were cohabiting (aOR = 0.72, 95% CI: 0.59–0.89) and those previously married (aOR = 0.68, 95% CI: 0.53–0.86) had lower odds of self-reporting genital discharge than those who had never been in a union. Compared with women with no children, those with one (aOR = 0.79, 95% CI: 0.65–0.95), two (aOR = 0.72, 95% CI: 0.57–0.90), three (aOR = 0.71, 95% CI: 0.56–0.91), and four or more children (aOR = 0.67, 95% CI: 0.53–0.86) had lower odds of self-reporting genital discharge. Women residing in the Greater Accra (aOR = 0.73, 95% CI: 0.56–0.95), Volta (aOR = 0.67, 95% CI: 0.51–0.88), Bono (aOR = 0.73, 95% CI: 0.56–0.95), Bono East (aOR = 0.67, 95% CI: 0.52–0.87), Oti (aOR = 0.59, 95% CI: 0.45–0.78), North East (aOR = 0.74, 95% CI: 0.56–0.97), and Upper East (aOR = 0.44, 95% CI: 0.33–0.58) had lower odds of self-reporting genital discharge. Women who engaged in multiple sexual partnerships (aOR = 1.65, 95% CI: 1.24–2.19) had higher odds of self-reporting genital discharge compared with those without multiple sexual partnerships. In addition, women living in the Northern (aOR = 1.59, 95% CI: 1.26–2.01) and Ahafo (aOR = 1.53, 95% CI: 1.20–1.95) regions had higher odds of self-reporting genital discharge than those from the Western region.

#### Genital sore or ulcer.

Women who were working (aOR = 0.79, 95% CI: 0.66–0.94) had lower odds of self-reporting genital sore or ulcer compared to those who were not working. Also, women with one (aOR = 0.76, 95% CI: 0.59–0.99) and four or more (aOR = 0.68, 95% CI: 0.48–0.96) children had lower odds of self-reporting genital sore or ulcer. Women who first had sex at age 17 or older (aOR = 0.72, 95% CI: 0.61–0.85) also had lower odds of self-reporting genital sore or ulcer compared to those who first had sex at age 16 or younger. Compared with women from the Western region, those who lived in the Greater Accra (aOR = 0.24, 95% CI: 0.14–0.43) and Upper East (aOR = 0.61, 95% CI: 0.40–0.92) regions had lower odds of self-reporting genital sore or ulcer. However, women from the Northern (aOR = 3.57, 95% CI: 2.51–5.08), Savannah (aOR = 2.10, 95% CI: 1.43–3.09), and Ahafo (aOR = 1.74, 95% CI: 1.22–2.49) had higher odds of self-reporting genital sore or ulcer. Women who engaged in multiple sexual partnerships (aOR = 3.06, 95% CI: 2.18–4.29) had higher odds of self-reporting genital sore or ulcer compared with their counterparts without multiple sexual partnerships (Table 4).

## Discussion

We examined the prevalence of self-reported STIs and related symptoms and their associated factors among women in Ghana. Age, number of living children, having multiple sexual partnerships, age at first sex, region, marital status, and visiting a health facility in the past 12 months were the factors associated with self-reported STIs and related symptoms among women in Ghana. Working status, however, was associated only with genital sores or ulcers.

Approximately three in ten women self-reported any STIs and related symptoms. This prevalence is higher than earlier estimates from Ghana, where 2-6% of women self-reported STIs and related symptoms [12,24,25]. The prevalence of STIs and related symptoms in our study also exceeds estimates from other sub-Saharan African countries, such as 11.8% in Sierra Leone and 12.8% in Guinea, but is lower than the 30.9% reported in Liberia [24]. Our finding may reflect greater awareness about STIs and willingness to disclose these symptoms during interviews. Nonetheless, underreporting due to stigma and social desirability bias could lead to underestimation of the true burden [26–27]. The data was collected in 2022, after the peak of the COVID-19 pandemic. Sexual activities may have decreased during this period [28] but increased afterwards, along with related risk factors such as economic stress and mental health challenges [29]. These factors highlight the need to normalise sexual health discussions, promote routine STI screening and strengthen sexual health services during health emergencies.

Women aged 40–49 had lower odds of reporting STIs and related symptoms compared to those aged 15-19. Similar findings have been reported in previous studies [23,31]. Younger women tend to have a higher prevalence of STIs due to biological susceptibility and socio-behavioural factors, such as early sexual debut and multiple sexual partnerships [23,30–32]. Our finding may partly reflect limited access to accurate sexual health information and youth-friendly services, as well as cost barriers to effective sexual and reproductive health service use among young people in Ghana [33]. Older women may report fewer STIs and related symptoms due to more stable sexual relationships and differences in healthcare-seeking behaviour. These include a lower perceived risk, under-recognition, and underdiagnosis of infections within this age group, as well as broader factors such as accumulated life experience and increased exposure to health information. [34–35]. They may also have greater autonomy and financial resources, allowing them to access preventive care and reproductive health services more easily [36]. Conversely, younger women may face stigma, fear of judgment, and logistical challenges in accessing STI-related care [33,37].

Women with children had lower odds of reporting STIs and related symptoms compared to those without children. Contrary findings have been reported in Mali [9], suggesting potential contextual differences between these two countries. Women with children are more likely to utilise healthcare services, such as antenatal and postnatal care, which provide more opportunities for routine health screenings, early diagnosis, and treatment of STIs as well as reduced risk exposure [38–40]. The association between parity and STIs and related symptoms is complex and its likely to be influenced by factors such as age, marital status, and socioeconomic conditions. Further longitudinal research is needed to better understand the association between parity and self-reported STIs and related symptoms in Ghana.

In addition, working women in Ghana had lower odds of reporting genital sores or ulcers. This finding contrasts with a study among men in sub-Saharan Africa, which report a positive association between employment status and STI risk [41], suggesting potential gender differences in behavioural or healthcare access patterns. Being employed may enhance financial autonomy and access to healthcare services, including STI screening and treatment [36], and is often linked to higher education and wealth, which influence STIs prevention and healthcare-seeking behaviour [42].

Women who engaged in multiple sexual partnerships had higher odds of reporting STIs and related symptoms, consistent with previous studies [9,43]. This may reflect increased exposure to infection associated with higher number of sexual partners, as well as underlying socioeconomic vulnerabilities such as transactional sex and financial pressures that influence sexual behaviour [36,44]. In addition, cultural practices such as polygyny may further expand sexual networks and increase STI risk, while female genital mutilation may influence vulnerability through biological and healthcare-related pathways [45].

Moreover, women who initiated sexual activity at age 17 or older had lower odds of reporting STIs and related symptoms compared to those with earlier sexual debut. Our finding is consistent with studies conducted in Mali [9] and Ghana [46,47], which show that early sexual initiation is associated with higher STI risk due to increased engagement in high-risk sexual behaviours. Delayed sexual debut may reflect better access to sexual health information and safer sex behaviours, as supported by evidence that school-based sex education and later sexual initiation are associated with reduced STI risk [48].

Furthermore, women residing in the Ahafo, Northern, and Savannah regions were more likely to report STIs and related symptoms compared to those living in the Western region. Our finding could be influenced by factors such as poverty, inadequate healthcare infrastructure, limited access to sexual and reproductive health services, and cultural practices such as early marriage and polygyny, which are prevalent in the northern belt of Ghana [19,24]. Also, deeply rooted traditional norms and practices in the Northern part of Ghana [49] may have limited women’s sexual autonomy, thereby elevating their risk of reporting STIs and related symptoms.

Women who had visited a health facility in the past 12 months had higher odds of reporting STIs and related symptoms. This finding may reflect an increased care-seeking among symptomatic women rather than a higher true prevalence of infections. Evidence suggests that health-seeking for STIs and related symptoms is prevalent among women who possess higher socioeconomic status, higher education, and greater awareness of sexual health issues [36,42]. For example, data from the 2014 GDHS indicate that a substantial proportion of young women with STI symptoms sought treatment, particularly those aged 20–24 years and those with secondary education or higher [50]. Similarly, a study conducted in Accra shows that wealthier women are more likely to seek care for STI symptoms [51]. These patterns of care-seeking behaviour may partly explain the higher reporting observed among women who visited health facilities in this study.

### Policy and practice implications

This study highlights the need for comprehensive, age-appropriate sexual health education in Ghana, particularly targeting younger women who are at higher risk of STIs. Promoting safer sexual practices, including consistent condom use and delayed sexual debut, is essential given the strong association between multiple sexual partnerships and STIs. Regional variations in STI prevalence underscore the importance of context-specific interventions that address local barriers such as limited healthcare access and socio-cultural norms. Strengthening STI service delivery through improved screening, provider training, and patient education is critical to enhance early detection and treatment. Coordinated efforts involving policymakers, healthcare providers, and communities are necessary to reduce the burden of STIs and improve women’s sexual and reproductive health outcomes. Further research is needed to explore behavioural and healthcare system factors that influence STIs and related symptoms reporting.

### Strengths and limitations

A notable strength of this study resides in the utilisation of the 2022 GDHS, a nationally representative survey providing reliable data that reflects the experiences of women in Ghana. The 2022 GDHS follows a well-established methodology, enhancing the reliability and comparability of the findings. The survey includes numerous demographic, socioeconomic, and behavioural variables relevant to understanding the prevalence of STIs and their associated factors. However, our study has some limitations. Relying on self-reporting of STIs and related symptoms introduces the possibility of under- and over-reporting due to social stigma, recall bias, and social desirability bias. The 2022 GDHS does not incorporate biological testing to confirm STI diagnoses, which limits the accuracy of prevalence estimates. The sensitive nature of sexual behaviour and STIs may lead some respondents to withhold or misrepresent information. Unmeasured factors (e.g., disparities in healthcare access and cultural norms) could influence the observed associations between the exposures and the outcomes. The 2022 GDHS utilised a cross-sectional design, which restricts the study’s ability to establish causality between exposure variables and the prevalence of STIs and related symptoms. Additionally, the number of missing observations and their subsequent exclusion from the analysis could have affected the findings. Therefore, the inferences drawn from the study should be based on the results presented. Furthermore, the response options “don’t know” were recoded as “no” for the outcome variables. This approach may have introduced misclassification bias, as some respondents who were uncertain about their STI status or symptoms may have been incorrectly classified as not having an STI. Consequently, the prevalence of self-reported STIs and related symptoms may have been underestimated, and the observed associations may have been attenuated. Finally, because genital discharge and genital sore or ulcer may arise from causes other than STIs, the composite outcome may overestimate the burden of true STIs.

## Conclusion

STIs and related symptoms are prevalent among women in Ghana. The findings showed that self-reporting of STIs and related symptoms was associated with age, number of children, multiple sexual partnerships, age at first sexual intercourse, working status, region, marital status, and recent visit to a health facility. Our findings emphasise the need for targeted sexual health education and intervention programmes in Ghana. Strategies should focus on younger women, promote safe sexual practices, and address regional differences in healthcare access and STI prevalence. Further research is needed to examine the association between parity and STI reporting, as well as the social and cultural factors behind regional disparities.

## Abbreviations

aOR: adjusted odds ratio
CI: confidence interval
DHS: Demographic and Health Survey
GDHS: Ghana Demographic and Health Survey
STIs: sexually transmitted infections
MEASURE DHS: Monitoring and Evaluation to Assess and Use Results Demographic and Health Surveys
