# Supplementary material for: Self-reported sexually transmitted infections and related symptoms among women in Ghana
Source: PLoS One. 2026 Jun 24;21(6):e0348770. doi: 10.1371/journal.pone.0348770 (PMC13293445; doi:10.1371/journal.pone.0348770)
Supplement: Attachment — Submitted filename: Review comments _PONE-D-25-67170.docx [file pone.0348770.s001.docx]

**Review summary**

This manuscript addresses an important public health issue using nationally representative DHS data and applies appropriate statistical methods. The topic is relevant to PLOS ONE readership and aligns well with global STI research priorities. However, the manuscript would benefit from **stronger methodological transparency, clearer justification of variable construction, deeper interpretation of findings, and language/structural polishing**.

The manuscript could be suitable for publication with the following revisions.

**1. Title and Scope**

**Lines 1–2**

*Self-reported sexually transmitted infections and their symptoms among women in Ghana: Findings from a nationwide analysis*

**Issues**

- The title does not specify that data are from the **2022 GDHS**.
- “Their symptoms” is vague.

**Suggested Revision**

*Self-reported sexually transmitted infections and related symptoms among women in Ghana: Evidence from the 2022 Ghana Demographic and Health Survey*

**2. Abstract – Methods and Results Need Precision**

**a) Methods**

**Lines 31–37**

- Sampling design, weighting, and handling of complex survey design are not mentioned.
- The composite outcome needs justification.

**Suggested Addition**

The analysis accounted for sampling weights, clustering, and stratification using DHS survey commands.

**b) Results**

**Lines 42–51**

- Results mix multiple outcomes but emphasize only the composite in regression.
- Regions are grouped (Ahafo, Northern, Savannah) without explanation.

**Suggested Revision**
Clarify that regression results mainly refer to the **composite outcome**, and specify that Savannah was significant only in bivariate or multivariable (whichever is correct).

**c) Conclusion**

**Lines 52–57**

- The phrase “women with more children and those initiating sex later” is ambiguous.

**Suggested Revision**

Self-reported STIs were lower among older women, those currently working, women with children, and those initiating sexual activity at later ages.

**3. Introduction – Improve Flow and Accuracy**

**a) Global Burden Statistics**

**Lines 64–68**

“…374 million new infections… resulting in 2.5 million deaths annually”

This statement is inaccurate. WHO does **not** attribute 2.5 million deaths annually to curable STIs.

**Suggested Action**
Verify and correct mortality statistics or remove death estimate.

**b) Redundancy**

**Lines 27–30 and 77–83**

Both describe global burden and surveillance limitations. Consider merging to avoid repetition.

**c) Research Gap**

**Lines 96–104**

The gap is mentioned but not strongly articulated.

**Suggested Addition**

To our knowledge, no recent nationally representative study has examined self-reported STIs and related symptoms among women using the 2022 GDHS, particularly incorporating regional disaggregation and behavioural factors.

**4. Methods – Clarify Key Analytical Decisions**

**a) Inclusion Criteria**

**Lines 122–124**

“women with data on all variables of interest”

**Concern**
This implies listwise deletion, which may bias estimates.

**Suggested Revision**

- Specify percentage excluded.
- Consider multiple imputation or justify complete-case analysis.

**b) Outcome Variable Coding**

**Lines 131–134**

“don’t know” recoded as “no”

**Concern**
This may underestimate prevalence.

**Suggested Revision**
Justify this choice or conduct sensitivity analysis excluding “don’t know”.

**c) Composite Outcome**

**Lines 126–127**

Explain why combining STI, discharge, and sores is epidemiologically meaningful.

**Suggested Addition**

A composite outcome was created to capture women experiencing any indication of STI or related symptoms, improving sensitivity in identifying potential infections in settings where laboratory confirmation is unavailable.

**d) Multicollinearity**

No mention of checking multicollinearity.

**Suggested Addition (Methods)**

Variance inflation factors (VIF) were assessed to examine multicollinearity.

**5. Results – Presentation Issues**

**a) Table 1**

**Line 166**

“61.% of the women…”

Typographical error.

**Correction**

61.0%

**b) Figure 1**

**Lines 171–177**

- No confidence intervals shown.
- Figure not described sufficiently.

**Suggested Improvement**
Include error bars or state in caption:

Bars represent weighted prevalence; error bars indicate 95% CI.

**c) Bivariate Tables**

**Tables 2 (Lines 197–203)**

- Two tables both labeled “Table 2”.
- Rename second as **Table 3** and shift subsequent tables.

**6. Multivariable Analysis – Interpretation**

**a) Health Facility Visit**

**Lines 225–227**

Interpretation is brief.

**Suggested Expansion**

This may reflect reverse causality, where women seek care due to symptoms, or increased detection among facility users rather than increased risk.

**b) Condom Use Associated with Higher STI Reporting**

(Table 3)

This counterintuitive result requires discussion.

**Suggested Explanation**

Women with symptoms may initiate condom use after infection, or socially desirable reporting may bias responses.

**c) Age Not Significant for “Any STI” Outcome**

(Table 3)

Yet emphasized in text.

**Suggested Action**
Differentiate which outcomes show age effects.

**7. Discussion – Needs Strengthening**

Currently limited interpretation is provided.

Add:

- Comparison with Ghana DHS 2014/2017.
- Comparison with Ethiopia, Mali, Nigeria studies.
- Policy implications (screening, youth-focused services, region-targeted interventions).

**8. Limitations Section Missing**

Add a dedicated subsection.

**Suggested Content**

- Self-reported data → recall/social desirability bias
- Cross-sectional design → no causality
- Symptoms not laboratory-confirmed
- Exclusion due to missing data

**9. Ethics Statement**

**Lines 155–158**

Should mention specific ethics committees and approval numbers if available.

**🟡 Minor Comments**

- Standardize terminology: use **STIs** consistently (avoid alternating with STDs).
- Italicize statistical symbols (p, OR).
- Replace “Genital sore or ulcer” with “genital sores/ulcers” throughout.
- Check references formatting consistency.
- Capitalization: “Islamic” → “Islam”.

**✅ Recommendation**

**Minor Revision**

The manuscript is methodologically sound but requires:

✔ Clearer justification of analytic choices
✔ Stronger interpretation
✔ Correction of technical and typographical issues
✔ Addition of limitations and expanded discussion
