# Supplementary material for: Self-reported sexually transmitted infections and related symptoms among women in Ghana
Source: PLoS One. 2026 Jun 24;21(6):e0348770. doi: 10.1371/journal.pone.0348770 (PMC13293445; doi:10.1371/journal.pone.0348770)
Supplement: Attachment — Submitted filename: Response to Reviewers Comments on STIs_R1 (1).docx [file pone.0348770.s002.docx]

**RESPONSE TO REVIEWERS’ COMMENTS**

**REVIEWER #1:**

Thank you for this wonderful well thought out manuscript. Its results will go a long way in informing the body of public health and STI medicine. appropriate methods were employed to adduce sound deductions.
However, some of the suggestions to address are as follows:

Response: Thank you.

1. Line 159-169 Results narrative. The narrative captured only percentages, would it be better if the absolute figures were also included alongside the percentages?

Response: Thank you. We have added the figures to the percentages.

2. Line 170-178 Prevalence. would it make it better to state the prevalence starting with the highest to the lowest?

Response: Thank you. We have revised the results description to list them from highest to lowest.

3. Line 179-220 Analysis. The final model at multivariate analysis has many variables which will affect the statistical output of the model.
At bivariate analysis e.g. religion didn't have a statistically significant P value, what would be the justification of having it in our final model at multivariate analysis?
There is visible overlapping of strata confidence intervals. it would be prudent to keenly analyze for effect modification and confounding which will eventually give as a better predictive model.

Response: Thank you. We included religion in the multivariate regression because one of its categories was statistically significant. Also, we checked for evidence of collinearity among the variables and found no evidence of high collinearity.

Conclusively its a privilege to have read and critiqued this manuscript. Great work team!!

Response: Thank you.

**REVIEWER 2**

Review summary

This manuscript addresses an important public health issue using nationally representative DHS data and applies appropriate statistical methods. The topic is relevant to PLOS ONE readership and aligns well with global STI research priorities. However, the manuscript would benefit from stronger methodological transparency, clearer justification of variable construction, deeper interpretation of findings, and language/structural polishing.

The manuscript could be suitable for publication with the following revisions.

1. Title and Scope

Lines 1–2: Self-reported sexually transmitted infections and their symptoms among women in Ghana: Findings from a nationwide analysis

Issues

- The title does not specify that data are from the 2022 GDHS.
- “Their symptoms” is vague.

Suggested Revision

Self-reported sexually transmitted infections and related symptoms among women in Ghana: Evidence from the 2022 Ghana Demographic and Health Survey

Response: The authors have revised the title to read “Self-reported sexually transmitted infections and related symptoms among women in Ghana: Evidence from the 2022 Ghana Demographic and Health Survey”.

2. Abstract – Methods and Results Need Precision

a) Methods

Lines 31–37

- Sampling design, weighting, and handling of complex survey design are not mentioned.
- The composite outcome needs justification.

Suggested Addition

The analysis accounted for sampling weights, clustering, and stratification using DHS survey commands.

Response: The authors have added the sentence that reads “The analysis accounted for sampling weights, clustering, and stratification using DHS survey commands” to the methods section.

b) Results

Lines 42–51

- Results mix multiple outcomes but emphasize only the composite in regression.
- Regions are grouped (Ahafo, Northern, Savannah) without explanation.

Suggested Revision
Clarify that regression results mainly refer to the composite outcome, and specify that Savannah was significant only in bivariate or multivariable (whichever is correct).

Response: Thank you for this comment. We have improved the description of the results section in the abstract to enhance clarity.

c) Conclusion

Lines 52–57

- The phrase “women with more children and those initiating sex later” is ambiguous.

Suggested Revision

Self-reported STIs were lower among older women, those currently working, women with children, and those initiating sexual activity at later ages.

Response: The authors have revised the sentence to read “Self-reported STIs were lower among older women, those currently working, women with children, and those initiating sexual activity at later ages”.

3. Introduction – Improve Flow and Accuracy

a) Global Burden Statistics

Lines 64–68

“…374 million new infections… resulting in 2.5 million deaths annually”

This statement is inaccurate. WHO does not attribute 2.5 million deaths annually to curable STIs.

Suggested Action
Verify and correct mortality statistics or remove death estimate.

Response: We have corrected the statistics.

b) Redundancy

Lines 27–30 and 77–83

Both describe global burden and surveillance limitations. Consider merging to avoid repetition.

Response: Thank you. We have revised the paragraphs.

c) Research Gap

Lines 96–104

The gap is mentioned but not strongly articulated.

Suggested Addition

To our knowledge, no recent nationally representative study has examined self-reported STIs and related symptoms among women using the 2022 GDHS, particularly incorporating regional disaggregation and behavioural factors.

Response: Thank you. We have added this suggestion to the study.

**4. Methods – Clarify Key Analytical Decisions**

a) Inclusion Criteria

Lines 122–124

“women with data on all variables of interest”

Concern
This implies listwise deletion, which may bias estimates.

Suggested Revision

- Specify percentage excluded.
- Consider multiple imputation or justify complete-case analysis.

Response: The authors have indicated that 13.4% of the 15,014 women of reproductive age were excluded from the study due to missing observations for one or more variables of interest.

b) Outcome Variable Coding

Lines 131–134

“don’t know” recoded as “no”

Concern
This may underestimate prevalence.

Suggested Revision
Justify this choice or conduct sensitivity analysis excluding “don’t know”.

Response: The don’t know category was less than 5%. Additionally, this category ‘don’t know’ had no variation in the prevalence estimate from the initial sensitivity test.

c) Composite Outcome

Lines 126–127

Explain why combining STI, discharge, and sores is epidemiologically meaningful.

Suggested Addition

A composite outcome was created to capture women experiencing any indication of STI or related symptoms, improving sensitivity in identifying potential infections in settings where laboratory confirmation is unavailable.

Response: Thank you for the suggestion. The authors have incorporated it into the study.

d) Multicollinearity

No mention of checking multicollinearity.

Suggested Addition (Methods)

Variance inflation factors (VIF) were assessed to examine multicollinearity.

Response: We have added that the variance inflation factor was used to examine evidence of multicollinearity among the variables.

5. Results – Presentation Issues

a) Table 1

Line 166

“61.% of the women…”

Typographical error.

Correction

61.0%

Response: We have corrected this to read ’61.0%’.

b) Figure 1

Lines 171–177

- No confidence intervals shown.
- Figure not described sufficiently.

Suggested Improvement
Include error bars or state in caption:

Bars represent weighted prevalence; error bars indicate 95% CI.

Response: We have revised Figure 1 to include the error bars.

c) Bivariate Tables

Tables 2 (Lines 197–203)

- Two tables both labeled “Table 2”.
- Rename second as Table 3 and shift subsequent tables.

Response: We have renamed the tables.

6. Multivariable Analysis – Interpretation

a) Health Facility Visit

Lines 225–227

Interpretation is brief.

Suggested Expansion

This may reflect reverse causality, where women seek care due to symptoms, or increased detection among facility users rather than increased risk.

Response: Thank you. We have included this interpretation in the discussion section.

b) Condom Use Associated with Higher STI Reporting

(Table 3)

This counterintuitive result requires discussion.

Suggested Explanation

Women with symptoms may initiate condom use after infection, or socially desirable reporting may bias responses.

Response: Thank you. However, the authors did not find any significant association between condom use and reporting any STIs and related symptoms.

c) Age Not Significant for “Any STI” Outcome

(Table 3)

Yet emphasized in text.

Suggested Action
Differentiate which outcomes show age effects.

Response: We have revised the results description for age to read “Women aged 40–44 years (aOR = 0.70; 95% CI: 0.52–0.93) and 45–49 years (aOR = 0.55; 95% CI: 0.40–0.76) were less likely to report any STIs and related symptoms compared with those aged 15-19. Similarly, lower likelihood of reporting genital discharge was found among women aged 40-44 (aOR = 0.69; 95% CI: 0.51, 0.95) and 45-49 (aOR = 0.52; 95% CI: 0.37, 0.74).

7. Discussion – Needs Strengthening

Currently limited interpretation is provided.

Add:

- Comparison with Ghana DHS 2014/2017.
- Comparison with Ethiopia, Mali, Nigeria studies.
- Policy implications (screening, youth-focused services, region-targeted interventions).

Response: Thank you. We have revised the policy implications section. Also, the current discussion section compared studies that used the DHS dataset and other studies from sub-Saharan African countries.

8. Limitations Section Missing

Add a dedicated subsection.

Suggested Content

- Self-reported data → recall/social desirability bias
- Cross-sectional design → no causality
- Symptoms not laboratory-confirmed
- Exclusion due to missing data

Response: Thank you. The authors have revised the limitations of the study to include the self-reported data, cross-sectional design, symptoms not laboratory-confirmed and exclusion of respondents with missing data.

9. Ethics Statement

Lines 155–158

Should mention specific ethics committees and approval numbers if available.

Response: We have added the institutional review board that granted ethics approval, with the approval number.

🟡 Minor Comments

- Standardize terminology: use STIs consistently (avoid alternating with STDs).
- Italicize statistical symbols (p, OR).
- Replace “Genital sore or ulcer” with “genital sores/ulcers” throughout.
- Check references formatting consistency.
- Capitalization: “Islamic” → “Islam”.

Response: We have corrected these minor errors throughout the manuscript.

✅ Recommendation

Minor Revision

The manuscript is methodologically sound but requires:

✔ Clearer justification of analytic choices
✔ Stronger interpretation
✔ Correction of technical and typographical issues
✔ Addition of limitations and expanded discussion

Response: Thank you. The authors have extensively revised the manuscript.

1. Can you please upload an additional copy of your revised manuscript that does not contain any tracked changes or highlighting as your main article file.  This will be used in the production process if your manuscript is accepted.  Please amend the file type for the file showing your changes to Revised Manuscript w/tracked changes. Please follow this link for more information: [http://blogs.PLOS.org/everyone/2011/05/10/how-to-submit-your-revised-manuscript/](http://blogs.plos.org/everyone/2011/05/10/how-to-submit-your-revised-manuscript/)

We have uploaded an additional copy of our manuscript that does not contain any tracked changes or highlighting in our article.

2. Please amend the title either on the online submission form or in your manuscript so that they are identical.

We have amended the title on the online submission form.

3. Thank you for your response. We still require accession numbers or specifics regarding the dataset you used so that interested researchers may search up your exact datasets that have been used. Currently, the links do not lead directly to the datasets and instead lead to the homepages of the websites.

We have further explain how interested researchers may search up our exact dataset that have been used. Please find it below:

The data underlying the results presented in this study are third-party data from the DHS Program. This study used data from the 2022 GDHS. The authors did not have special access privileges. The data are available upon request through registration with the DHS Program at <https://dhsprogram.com/data/>. The specific dataset used in this study can be accessed at: <https://dhsprogram.com/data/dataset/Ghana_Standard-DHS_2022.cfm?flag=1>
