# Supplementary material for: Self-reported sexually transmitted infections and related symptoms among women in Ghana
Source: PLoS One. 2026 Jun 24;21(6):e0348770. doi: 10.1371/journal.pone.0348770 (PMC13293445; doi:10.1371/journal.pone.0348770)
Supplement: Attachment — Submitted filename: Review report.odt [file pone.0348770.s003.odt]

# PONE-D-25-67170_R1
## Overall Assessment
I have carefully reviewed the revised manuscript "Self-reported sexually transmitted infections and related symptoms among women in Ghana: Evidence from the 2022 Ghana Demographic and Health Survey" and the authors' responses to my previous review comments. The authors have made substantial and thoughtful revisions that have significantly improved the manuscript. All major concerns raised in my initial review have been adequately addressed. The manuscript is now methodologically transparent, well-structured, and provides meaningful interpretation of findings.
## Response to Previous Comments
### 1. Title and Scope ✓ ADDRESSED
The authors have revised the title to specify the 2022 GDHS and changed "their symptoms" to "related symptoms," which is more precise.
### 2. Abstract ✓ ADDRESSED
Methods: Added statement about accounting for sampling weights, clustering, and stratification
Results: Improved clarity regarding composite outcome and regional findings
Conclusion: Revised ambiguous phrasing about women with children
### 3. Introduction ✓ ADDRESSED
Corrected mortality statistics (now accurately reflects WHO data)
Merged redundant paragraphs on global burden and surveillance limitations
Added clear research gap statement referencing 2022 GDHS
### 4. Methods ✓ ADDRESSED
Concern
Authors’ response
Inclusion criteria/listwise deletion
Authors now specify 13.4% excluded; justify complete-case analysis
"Don't know" recoded as "no"
Justified with <5% missing and sensitivity test results
Composite outcome justification
Added explanatory sentence as suggested
Multicollinearity
Added VIF assessment statement
### 5. Results ✓ ADDRESSED
Table 1: Corrected "61. %" to "61.0%"
Figure 1: Added error bars representing 95% CIs
Bivariate tables: Correctly renamed as Table 2 and Table 3
### 6. Multivariable Analysis Interpretation ✓ ADDRESSED
Health facility visits: Added reverse causality explanation in discussion
Condom use: Correctly noted no significant association found
Age effects: Differentiated which outcomes show significance
### 7. Discussion ✓ ADDRESSED
Added comparisons with previous GDHS rounds (2014) and studies from Ethiopia, Mali, and other sub-Saharan African countries. Policy implications section substantially expanded.
### 8. Limitations ✓ ADDRESSED
New dedicated subsection includes:
Self-report bias (recall/social desirability)
Cross-sectional design (no causality)
Lack of laboratory confirmation
Missing data exclusion
### 9. Ethics Statement ✓ ADDRESSED
Added ICF Institutional Review Board reference number (45 CFR 46) and Ghana Health Service Ethics Review Committee approval.
### Minor Comments ✓ ADDRESSED
Standardized STI terminology
Italicized statistical symbols
Changed to "genital sores/ulcers" throughout
Capitalized "Islam"
## 
## Final recommendation
The manuscript is now suitable for publication. The authors have responded comprehensively to all reviewer concerns, strengthened the methodological rigor, improved interpretation of findings, and addressed all technical and typographical issues. The study makes a valuable contribution to understanding STI burden among women in Ghana using nationally representative data.
