# Supplementary material for: Self-reported sexually transmitted infections and related symptoms among women in Ghana
Source: PLoS One. 2026 Jun 24;21(6):e0348770. doi: 10.1371/journal.pone.0348770 (PMC13293445; doi:10.1371/journal.pone.0348770)
Supplement: Peer review history

# Peer review: Decision Letter 0

**DOI:** 10.1371/journal.pone.0348770.r001

**Authors:** Kahsu Gebrekidan

2 Feb 2026

PONE-D-25-67170

Self-reported sexually transmitted infections and their symptoms among women in Ghana: Findings from a nationwide analysis

PLOS One

Dear Dr. Wongnaah,

Thank you for submitting your manuscript to PLOS ONE. After careful consideration, we feel that it has merit but does not fully meet PLOS ONE’s publication criteria as it currently stands. Therefore, we invite you to submit a revised version of the manuscript that addresses the points raised during the review process.

Please submit your revised manuscript by Mar 19 2026 11:59PM. If you will need more time than this to complete your revisions, please reply to this message or contact the journal office at plosone@plos.org. When you're ready to submit your revision, log on to https://www.editorialmanager.com/pone/ and select the 'Submissions Needing Revision' folder to locate your manuscript file.

Please include the following items when submitting your revised manuscript:

- A letter that responds to each point raised by the academic editor and reviewer(s). You should upload this letter as a separate file labeled 'Response to Reviewers'.
- A marked-up copy of your manuscript that highlights changes made to the original version. You should upload this as a separate file labeled 'Revised Manuscript with Track Changes'.
- An unmarked version of your revised paper without tracked changes. You should upload this as a separate file labeled 'Manuscript'.

-->If you would like to make changes to your financial disclosure, please include your updated statement in your cover letter. Guidelines for resubmitting your figure files are available below the reviewer comments at the end of this letter.

If applicable, we recommend that you deposit your laboratory protocols in protocols.io to enhance the reproducibility of your results. Protocols.io assigns your protocol its own identifier (DOI) so that it can be cited independently in the future. For instructions see: https://journals.plos.org/plosone/s/submission-guidelines#loc-laboratory-protocols. Additionally, PLOS ONE offers an option for publishing peer-reviewed Lab Protocol articles, which describe protocols hosted on protocols.io. Read more information on sharing protocols at https://plos.org/protocols?utm_medium=editorial-email&utm_source=authorletters&utm_campaign=protocols.

We look forward to receiving your revised manuscript.

Kind regards,

Kahsu Gebrekidan, Ph.D.

Academic Editor

PLOS One

Journal Requirements:

When submitting your revision, we need you to address these additional requirements.

1. Please ensure that your manuscript meets PLOS ONE's style requirements, including those for file naming. The PLOS ONE style templates can be found at

https://journals.plos.org/plosone/s/file?id=wjVg/PLOSOne_formatting_sample_main_body.pdf and

https://journals.plos.org/plosone/s/file?id=ba62/PLOSOne_formatting_sample_title_authors_affiliations.pdf

2. In the online submission form, you indicated that “The data underlying the findings of this study are publicly available from the Demographic and Health Surveys (DHS) Program. The datasets can be accessed upon free registration and request via the DHS Program website (https://dhsprogram.com”

All PLOS journals now require all data underlying the findings described in their manuscript to be freely available to other researchers, either 1. In a public repository, 2. Within the manuscript itself, or 3. Uploaded as supplementary information.

This policy applies to all data except where public deposition would breach compliance with the protocol approved by your research ethics board. If your data cannot be made publicly available for ethical or legal reasons (e.g., public availability would compromise patient privacy), please explain your reasons on resubmission and your exemption request will be escalated for approval.

3. Please include your full ethics statement in the ‘Methods’ section of your manuscript file. In your statement, please include the full name of the IRB or ethics committee who approved or waived your study, as well as whether or not you obtained informed written or verbal consent. If consent was waived for your study, please include this information in your statement as well.

4. If the reviewer comments include a recommendation to cite specific previously published works, please review and evaluate these publications to determine whether they are relevant and should be cited. There is no requirement to cite these works unless the editor has indicated otherwise.

5. Please review your reference list to ensure that it is complete and correct. If you have cited papers that have been retracted, please include the rationale for doing so in the manuscript text, or remove these references and replace them with relevant current references. Any changes to the reference list should be mentioned in the rebuttal letter that accompanies your revised manuscript. If you need to cite a retracted article, indicate the article’s retracted status in the References list and also include a citation and full reference for the retraction notice.

[Note: HTML markup is below. Please do not edit.]

Reviewers' comments:

Reviewer's Responses to Questions

**Comments to the Author**

1. Is the manuscript technically sound, and do the data support the conclusions?

The manuscript must describe a technically sound piece of scientific research with data that supports the conclusions. Experiments must have been conducted rigorously, with appropriate controls, replication, and sample sizes. The conclusions must be drawn appropriately based on the data presented.

Reviewer #1: Yes

Reviewer #2: Yes

**********

2. Has the statistical analysis been performed appropriately and rigorously?

Reviewer #1: Yes

Reviewer #2: Yes

**********

3. Have the authors made all data underlying the findings in their manuscript fully available?

The PLOS Data policy requires authors to make all data underlying the findings described in their manuscript fully available without restriction, with rare exception (please refer to the Data Availability Statement in the manuscript PDF file). The data should be provided as part of the manuscript or its supporting information, or deposited to a public repository. For example, in addition to summary statistics, the data points behind means, medians and variance measures should be available. If there are restrictions on publicly sharing data—e.g. participant privacy or use of data from a third party—those must be specified.

Reviewer #1: Yes

Reviewer #2: Yes

**********

4. Is the manuscript presented in an intelligible fashion and written in standard English?

PLOS ONE does not copyedit accepted manuscripts, so the language in submitted articles must be clear, correct, and unambiguous. Any typographical or grammatical errors should be corrected at revision, so please note any specific errors here.

Reviewer #1: Yes

Reviewer #2: Yes

**********

5. Review Comments to the Author

Please use the space provided to explain your answers to the questions above. You may also include additional comments for the author, including concerns about dual publication, research ethics, or publication ethics. (Please upload your review as an attachment if it exceeds 20,000 characters)

Reviewer #1: Thankyou for this wonderful well thought out manuscript. Its results will go a long way in informing the body of public health and STI medicine. appropriate methods were employed to adduce sound deductions.

However, some of the suggestions to address are as follows:

1. Line 159-169 Results narrative. The narrative captured only percentages, would it be better if the absolute figures were also included alongside the percentages?

2. Line 170-178 Prevalence. would it make it better to state the prevalence starting with the highest to the lowest?

3. Line 179-220 Analysis. The final model at multivariate analysis has many variables which will affect the statistical output of the model.

At bivariate analysis e.g. religion didn't have a statistically significant P value, what would be the justification of having it in our final model at multivariate analysis?

There is visible overlapping of strata confidence intervals. it would be prudent to keenly analyze for effect modification and confounding which will eventually give as a better predictive model.

conclusively its a privilege to have read and critiqued this manuscript. Great work team!!

Reviewer #2: This manuscript addresses an important public health issue using nationally representative DHS data and applies appropriate statistical methods. The topic is relevant to PLOS ONE readership and aligns well with global STI research priorities. However, the manuscript would benefit from stronger methodological transparency, clearer justification of variable construction, deeper interpretation of findings, and language/structural polishing.

**********

6. PLOS authors have the option to publish the peer review history of their article (what does this mean?). If published, this will include your full peer review and any attached files.

If you choose “no”, your identity will remain anonymous but your review may still be made public.

**Do you want your identity to be public for this peer review?**   For information about this choice, including consent withdrawal, please see our Privacy Policy.

Reviewer #1: **Yes:**   Dr Aeku Job Michael

Reviewer #2: No

**********

[NOTE: If reviewer comments were submitted as an attachment file, they will be attached to this email and accessible via the submission site. Please log into your account, locate the manuscript record, and check for the action link "View Attachments". If this link does not appear, there are no attachment files.]

To ensure your figures meet our technical requirements, please review our figure guidelines: https://journals.plos.org/plosone/s/figures

You may also use PLOS’s free figure tool, NAAS, to help you prepare publication quality figures: https://journals.plos.org/plosone/s/figures#loc-tools-for-figure-preparation.

NAAS will assess whether your figures meet our technical requirements by comparing each figure against our figure specifications.

Attachment Submitted filename: Review comments _PONE-D-25-67170.docx

# Author comment: Author response to Decision Letter 1

**DOI:** 10.1371/journal.pone.0348770.r002

5 Apr 2026

RESPONSE TO REVIEWERS’ COMMENTS

REVIEWER #1:

Thank you for this wonderful well thought out manuscript. Its results will go a long way in informing the body of public health and STI medicine. appropriate methods were employed to adduce sound deductions.

However, some of the suggestions to address are as follows:

Response: Thank you.

1. Line 159-169 Results narrative. The narrative captured only percentages, would it be better if the absolute figures were also included alongside the percentages?

Response: Thank you. We have added the figures to the percentages.

2. Line 170-178 Prevalence. would it make it better to state the prevalence starting with the highest to the lowest?

Response: Thank you. We have revised the results description to list them from highest to lowest.

3. Line 179-220 Analysis. The final model at multivariate analysis has many variables which will affect the statistical output of the model.

At bivariate analysis e.g. religion didn't have a statistically significant P value, what would be the justification of having it in our final model at multivariate analysis?

There is visible overlapping of strata confidence intervals. it would be prudent to keenly analyze for effect modification and confounding which will eventually give as a better predictive model.

Response: Thank you. We included religion in the multivariate regression because one of its categories was statistically significant. Also, we checked for evidence of collinearity among the variables and found no evidence of high collinearity.

Conclusively its a privilege to have read and critiqued this manuscript. Great work team!!

Response: Thank you.

REVIEWER 2

Review summary

This manuscript addresses an important public health issue using nationally representative DHS data and applies appropriate statistical methods. The topic is relevant to PLOS ONE readership and aligns well with global STI research priorities. However, the manuscript would benefit from stronger methodological transparency, clearer justification of variable construction, deeper interpretation of findings, and language/structural polishing.

The manuscript could be suitable for publication with the following revisions.

1. Title and Scope

Lines 1–2: Self-reported sexually transmitted infections and their symptoms among women in Ghana: Findings from a nationwide analysis

Issues

• The title does not specify that data are from the 2022 GDHS.

• “Their symptoms” is vague.

Suggested Revision

Self-reported sexually transmitted infections and related symptoms among women in Ghana: Evidence from the 2022 Ghana Demographic and Health Survey

Response: The authors have revised the title to read “Self-reported sexually transmitted infections and related symptoms among women in Ghana: Evidence from the 2022 Ghana Demographic and Health Survey”.

2. Abstract – Methods and Results Need Precision

a) Methods

Lines 31–37

• Sampling design, weighting, and handling of complex survey design are not mentioned.

• The composite outcome needs justification.

Suggested Addition

The analysis accounted for sampling weights, clustering, and stratification using DHS survey commands.

Response: The authors have added the sentence that reads “The analysis accounted for sampling weights, clustering, and stratification using DHS survey commands” to the methods section.

b) Results

Lines 42–51

• Results mix multiple outcomes but emphasize only the composite in regression.

• Regions are grouped (Ahafo, Northern, Savannah) without explanation.

Suggested Revision

Clarify that regression results mainly refer to the composite outcome, and specify that Savannah was significant only in bivariate or multivariable (whichever is correct).

Response: Thank you for this comment. We have improved the description of the results section in the abstract to enhance clarity.

c) Conclusion

Lines 52–57

• The phrase “women with more children and those initiating sex later” is ambiguous.

Suggested Revision

Self-reported STIs were lower among older women, those currently working, women with children, and those initiating sexual activity at later ages.

Response: The authors have revised the sentence to read “Self-reported STIs were lower among older women, those currently working, women with children, and those initiating sexual activity at later ages”.

3. Introduction – Improve Flow and Accuracy

a) Global Burden Statistics

Lines 64–68

“…374 million new infections… resulting in 2.5 million deaths annually”

This statement is inaccurate. WHO does not attribute 2.5 million deaths annually to curable STIs.

Suggested Action

Verify and correct mortality statistics or remove death estimate.

Response: We have corrected the statistics.

b) Redundancy

Lines 27–30 and 77–83

Both describe global burden and surveillance limitations. Consider merging to avoid repetition.

Response: Thank you. We have revised the paragraphs.

c) Research Gap

Lines 96–104

The gap is mentioned but not strongly articulated.

Suggested Addition

To our knowledge, no recent nationally representative study has examined self-reported STIs and related symptoms among women using the 2022 GDHS, particularly incorporating regional disaggregation and behavioural factors.

Response: Thank you. We have added this suggestion to the study.

4. Methods – Clarify Key Analytical Decisions

a) Inclusion Criteria

Lines 122–124

“women with data on all variables of interest”

Concern

This implies listwise deletion, which may bias estimates.

Suggested Revision

• Specify percentage excluded.

• Consider multiple imputation or justify complete-case analysis.

Response: The authors have indicated that 13.4% of the 15,014 women of reproductive age were excluded from the study due to missing observations for one or more variables of interest.

b) Outcome Variable Coding

Lines 131–134

“don’t know” recoded as “no”

Concern

This may underestimate prevalence.

Suggested Revision

Justify this choice or conduct sensitivity analysis excluding “don’t know”.

Response: The don’t know category was less than 5%. Additionally, this category ‘don’t know’ had no variation in the prevalence estimate from the initial sensitivity test.

c) Composite Outcome

Lines 126–127

Explain why combining STI, discharge, and sores is epidemiologically meaningful.

Suggested Addition

A composite outcome was created to capture women experiencing any indication of STI or related symptoms, improving sensitivity in identifying potential infections in settings where laboratory confirmation is unavailable.

Response: Thank you for the suggestion. The authors have incorporated it into the study.

d) Multicollinearity

No mention of checking multicollinearity.

Suggested Addition (Methods)

Variance inflation factors (VIF) were assessed to examine multicollinearity.

Response: We have added that the variance inflation factor was used to examine evidence of multicollinearity among the variables.

5. Results – Presentation Issues

a) Table 1

Line 166

“61.% of the women…”

Typographical error.

Correction

61.0%

Response: We have corrected this to read ’61.0%’.

b) Figure 1

Lines 171–177

• No confidence intervals shown.

• Figure not described sufficiently.

Suggested Improvement

Include error bars or state in caption:

Bars represent weighted prevalence; error bars indicate 95% CI.

Response: We have revised Figure 1 to include the error bars.

c) Bivariate Tables

Tables 2 (Lines 197–203)

• Two tables both labeled “Table 2”.

• Rename second as Table 3 and shift subsequent tables.

Response: We have renamed the tables.

6. Multivariable Analysis – Interpretation

a) Health Facility Visit

Lines 225–227

Interpretation is brief.

Suggested Expansion

This may reflect reverse causality, where women seek care due to symptoms, or increased detection among facility users rather than increased risk.

Response: Thank you. We have included this interpretation in the discussion section.

b) Condom Use Associated with Higher STI Reporting

(Table 3)

This counterintuitive result requires discussion.

Suggested Explanation

Women with symptoms may initiate condom use after infection, or socially desirable reporting may bias responses.

Response: Thank you. However, the authors did not find any significant association between condom use and reporting any STIs and related symptoms.

c) Age Not Significant for “Any STI” Outcome

(Table 3)

Yet emphasized in text.

Suggested Action

Differentiate which outcomes show age effects.

Response: We have revised the results description for age to read “Women aged 40–44 years (aOR = 0.70; 95% CI: 0.52–0.93) and 45–49 years (aOR = 0.55; 95% CI: 0.40–0.76) were less likely to report any STIs and related symptoms compared with those aged 15-19. Similarly, lower likelihood of reporting genital discharge was found among women aged 40-44 (aOR = 0.69; 95% CI: 0.51, 0.95) and 45-49 (aOR = 0.52; 95% CI: 0.37, 0.74).

7. Discussion – Needs Strengthening

Currently limited interpretation is provided.

Add:

• Comparison with Ghana DHS 2014/2017.

• Comparison with Ethiopia, Mali, Nigeria studies.

• Policy implications (screening, youth-focused services, region-targeted interventions).

Response: Thank you. We have revised the policy implications section. Also, the current discussion section compared studies that used the DHS dataset and other studies from sub-Saharan African countries.

8. Limitations Section Missing

Add a dedicated subsection.

Suggested Content

• Self-reported data → recall/social desirability bias

• Cross-sectional design → no causality

• Symptoms not laboratory-confirmed

• Exclusion due to missing data

Response: Thank you. The authors have revised the limitations of the study to include the self-reported data, cross-sectional design, symptoms not laboratory-confirmed and exclusion of respondents with missing data.

9. Ethics Statement

Lines 155–158

Should mention specific ethics committees and approval numbers if available.

Response: We have added the institutional review board that granted ethics approval, with the approval number.

• Minor Comments

• Standardize terminology: use STIs consistently (avoid alternating with STDs).

• Italicize statistical symbols (p, OR).

• Replace “Genital sore or ulcer” with “genital sores/ulcers” throughout.

• Check references formatting consistency.

• Capitalization: “Islamic” → “Islam”.

Response: We have corrected these minor errors throughout the manuscript.

✅ Recommendation

Minor Revision

The manuscript is methodologically sound but requires:

✔ Clearer justification of analytic choices

✔ Stronger interpretation

✔ Correction of technical and typographical issues

✔ Addition of limitations and expanded discussion

Response: Thank you. The authors have extensively revised the manuscript.

Attachment Submitted filename: Response to Reviewers Comments on STIs_R1 (1).docx

# Peer review: Decision Letter 1

**DOI:** 10.1371/journal.pone.0348770.r003

**Authors:** Kahsu Gebrekidan

22 Apr 2026

Self-reported sexually transmitted infections and related symptoms among women in Ghana: Evidence from the 2022 Ghana Demographic and Health Survey

PONE-D-25-67170R1

Dear Dr. Florence,

We’re pleased to inform you that your manuscript has been judged scientifically suitable for publication and will be formally accepted for publication once it meets all outstanding technical requirements.

Within one week, you’ll receive an e-mail detailing the required amendments. When these have been addressed, you’ll receive a formal acceptance letter and your manuscript will be scheduled for publication.

An invoice will be generated when your article is formally accepted. Please note, if your institution has a publishing partnership with PLOS and your article meets the relevant criteria, all or part of your publication costs will be covered. Please make sure your user information is up-to-date by logging into Editorial Manager at Editorial Manager® and clicking the ‘Update My Information' link at the top of the page. For questions related to billing, please contact billing support.

If your institution or institutions have a press office, please notify them about your upcoming paper to help maximize its impact. If they’ll be preparing press materials, please inform our press team as soon as possible -- no later than 48 hours after receiving the formal acceptance. Your manuscript will remain under strict press embargo until 2 pm Eastern Time on the date of publication. For more information, please contact onepress@plos.org.

Kind regards,

Kahsu Gebrekidan, Ph.D.

Academic Editor

PLOS One

Additional Editor Comments (optional):

Reviewers' comments:

Reviewer's Responses to Questions

**Comments to the Author**

1. If the authors have adequately addressed your comments raised in a previous round of review and you feel that this manuscript is now acceptable for publication, you may indicate that here to bypass the “Comments to the Author” section, enter your conflict of interest statement in the “Confidential to Editor” section, and submit your "Accept" recommendation.

Reviewer #1: All comments have been addressed

Reviewer #2: All comments have been addressed

**********

2. Is the manuscript technically sound, and do the data support the conclusions?

The manuscript must describe a technically sound piece of scientific research with data that supports the conclusions. Experiments must have been conducted rigorously, with appropriate controls, replication, and sample sizes. The conclusions must be drawn appropriately based on the data presented.

Reviewer #1: Yes

Reviewer #2: Yes

**********

3. Has the statistical analysis been performed appropriately and rigorously?

Reviewer #1: Yes

Reviewer #2: Yes

**********

4. Have the authors made all data underlying the findings in their manuscript fully available?

The PLOS Data policy requires authors to make all data underlying the findings described in their manuscript fully available without restriction, with rare exception (please refer to the Data Availability Statement in the manuscript PDF file). The data should be provided as part of the manuscript or its supporting information, or deposited to a public repository. For example, in addition to summary statistics, the data points behind means, medians and variance measures should be available. If there are restrictions on publicly sharing data—e.g. participant privacy or use of data from a third party—those must be specified.

Reviewer #1: Yes

Reviewer #2: Yes

**********

5. Is the manuscript presented in an intelligible fashion and written in standard English?

PLOS ONE does not copyedit accepted manuscripts, so the language in submitted articles must be clear, correct, and unambiguous. Any typographical or grammatical errors should be corrected at revision, so please note any specific errors here.

Reviewer #1: Yes

Reviewer #2: Yes

**********

6. Review Comments to the Author

Please use the space provided to explain your answers to the questions above. You may also include additional comments for the author, including concerns about dual publication, research ethics, or publication ethics. (Please upload your review as an attachment if it exceeds 20,000 characters)

Reviewer #1: The shared compliance report of the authors is in line with the issues raised during review. This enriches the manuscript and makes it better readable and informative.

Thankyou

Reviewer #2: The manuscript is now suitable for publication. The authors have responded comprehensively to all reviewer concerns, strengthened the methodological rigor, improved interpretation of findings, and addressed all technical and typographical issues. The study makes a valuable contribution to understanding STI burden among women in Ghana using nationally representative data.

**********

7. PLOS authors have the option to publish the peer review history of their article (what does this mean?). If published, this will include your full peer review and any attached files.

If you choose “no”, your identity will remain anonymous but your review may still be made public.

**Do you want your identity to be public for this peer review?**   For information about this choice, including consent withdrawal, please see our Privacy Policy.

Reviewer #1: **Yes:**   Aeku Job Michael

Reviewer #2: **Yes:**   Kibir Temesgen Assefa

**********

Attachment Submitted filename: Review report.odt

# Editor report: Acceptance letter

**DOI:** 10.1371/journal.pone.0348770.r004

**Authors:** Kahsu Gebrekidan

PONE-D-25-67170R1

PLOS One

Dear Dr. Wongnaah,

I'm pleased to inform you that your manuscript has been deemed suitable for publication in PLOS One. Congratulations! Your manuscript is now being handed over to our production team.

At this stage, our production department will prepare your paper for publication. This includes ensuring the following:

* All references, tables, and figures are properly cited

* All relevant supporting information is included in the manuscript submission,

* There are no issues that prevent the paper from being properly typeset

You will receive further instructions from the production team, including instructions on how to review your proof when it is ready. Please keep in mind that we are working through a large volume of accepted articles, so please give us a few days to review your paper and let you know the next and final steps.

Lastly, if your institution or institutions have a press office, please let them know about your upcoming paper now to help maximize its impact. If they'll be preparing press materials, please inform our press team within the next 48 hours. Your manuscript will remain under strict press embargo until 2 pm Eastern Time on the date of publication. For more information, please contact onepress@plos.org.

You will receive an invoice from PLOS for your publication fee after your manuscript has reached the completed accept phase. If you receive an email requesting payment before acceptance or for any other service, this may be a phishing scheme. Learn how to identify phishing emails and protect your accounts at https://explore.plos.org/phishing.

If we can help with anything else, please email us at customercare@plos.org.

Thank you for submitting your work to PLOS ONE and supporting open access.

Kind regards,

PLOS ONE Editorial Office Staff

on behalf of

Dr. Kahsu Gebrekidan

Academic Editor

PLOS One
